# How does variability of immune system genes affect placentation?

**DOI:** 10.1016/j.placenta.2011.05.001

**Published:** 2011-08

**Authors:** F. Colucci, S. Boulenouar, J. Kieckbusch, A. Moffett

**Affiliations:** a Department of Obstetrics and Gynaecology, University of Cambridge Clinical School, Box 223, The Rosie Hospital, Robinson Way, Cambridge CB2 0SW, UK; b NIHR Cambridge Comprehensive Biomedical Research Centre, UK; c Department of Pathology, University of Cambridge, Tennis Court Road, Cambridge CB2 1QP, UK; d Centre for Trophoblast Research, University of Cambridge, Downing Street, Cambridge, CB2 3EG, UK

**Keywords:** Uterine Natural Killer (uNK) cells, Trophoblast, Pregnancy, Major histocompatibility complex (MHC), Killer-cell immunoglobulin-like receptors (KIR), Ly49 receptors

## Abstract

Formation of the placenta is a crucial step in mammalian pregnancy. Apart from its function in ensuring an optimal supply of nutrients and oxygen to the fetus, the placenta is also the interface at which allo-recognition of invading trophoblast cells by the maternal immune system can potentially occur. We summarise here the “state of the art” on how variability of immune system genes that code for major histocompatibility complex (MHC) molecules and natural killer receptors (NKR) may impact on human placentation. MHC and NKR are the most polymorphic human genes. Our recent reports point out that specific combinations of fetal MHC and maternal NKR genes in humans correlate with the risk of pre-eclampsia, recurrent miscarriage (RM) and fetal growth restriction (FGR). Research in this field is still at an early stage and future studies in mouse and humans will be needed before the results can be translated to clinical applications. We discuss our recent work, as well as the opportunities offered by mouse genetics, to understand the cellular and molecular mechanisms underlying immune interactions at the maternal-fetal interface.

## 1 Introduction

Uterine natural killer (uNK) cells are the main maternal immune cells present in decidualised endometrium prior to and during the establishment of the placenta in species with invasive haemochorial placentation (including humans and mice) [1]. NK cells are part of the lymphoid lineage and like T and B lymphocytes can be divided in several subpopulations [2]. The NK cells present in the uterus are phenotypically and functionally unlike those present in the systemic circulation [3,4]. Their exact functions in pregnancy are unknown, but available evidence points to a role in regulating the complex process of placentation. In particular, they are thought to be involved in co-ordinating access of the placental trophoblast cells to the uterine arteries. Trophoblast transformation of the arteries results in high conductance vessels, ensuring an adequate supply of oxygen and nutrients to the feto-placental unit in both human and mouse [5–8].

Many of the genes selected during evolution to regulate reproduction are invariable. However, reproduction is the only natural situation in vertebrates where two genetically different individuals coexist. Therefore, the intrinsic variability of immune system genes may well be one of the key selective pressures shaping the evolution of highly invasive haemochorial placentation. Lymphocytes have a variety of receptors capable of discerning ligands present on unhealthy cells or on those from another individual and indeed, uNK cells do have an array of receptors capable of binding fetal trophoblast ligands [9–11]. One important set of ligands for NK cells are MHC class I molecules, some of which are expressed by invasive trophoblast [12]. MHC and NKR are both highly polymorphic gene systems and indeed they are unique in their extreme variability [13,14].

We have established that certain combinations of maternal NKR and fetal MHC genotypes are risk factors for common disorders of pregnancy in humans [15–18]. Understanding how ‘good’ or ‘bad’ NKR/MHC combinations translate into different NK cell functions and subsequently affect trophoblast invasion and arterial blood flow is now a major challenge. The human maternal-fetal interface is inaccessible in early pregnancy and it is impossible to determine at this stage the pregnancies that will result in a poor outcome. To surmount these difficulties in human pregnancy research, we have begun to explore the similarities and differences between human and mice NKR and potential trophoblast ligands with the aim of developing mouse models that will elucidate how NK cell–trophoblast interactions contribute to placentation.

## 2 Trophoblast and decidual NK cells

**Fig. 1:**
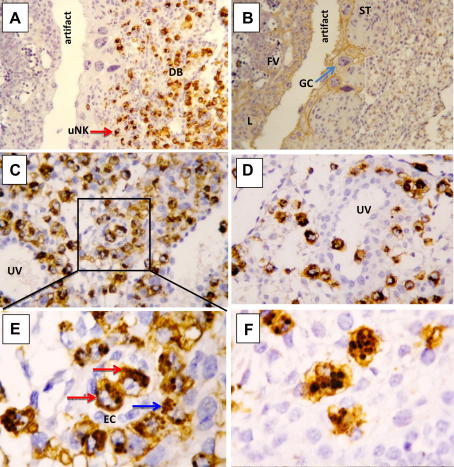
Immunohistochemistry of midsagittal sections of a mouse implantation site at gestational day 8.5. A) uNK cells, stained brown by Dolichos Biflorus Agglutinin (DBA) accumulate in the decida basalis (DB). The red arrow points to a DBA+ uNK cell; B) The adjacent section was stained with cytokeratin to identify trophoblast cells, including spongiotrophoblast (ST), invasive giant cells (GC, light blue arrow) and labirinth (L). Fetal vessels (FV) with nucleated red cells are also visible; C–D) The DBA+ uNK cells are associated with maternal uterine vessels (UV) and may participate in their remodelling; E) A higher magnification of the field in C) shows two intravascular DBA+ uNK cells (red arrows) within endothelial cells (EC) and several DBA+ uNK cells in the media (blue arrow); F) DBA+ uNK cells at this stage are large and with prominent granules.

The invasion of human extravillous trophoblast (EVT) into decidua with transformation of the spiral arteries is well documented [1,8]. This process is a critical determinant of reproductive outcome with defective invasion leading to major clinical problems in pregnancy, including pre-eclampsia [19], FGR [20,21] and RM [22,23]. The decidua basalis where the placenta implants is the main tissue site where cells from two individuals intermingle. Because the placental cells are semi-allogeneic, a reasonable hypothesis is that the maternal uterine immune cells regulate trophoblast behaviour. NK cells are present in great abundance at the site of placentation in both humans and mice [1] (Fig. 1). The transformation of the uterine mucosa from endometrium to decidua is characterised by the appearance of large numbers of distinctive NK cells not found elsewhere in the body [24]. Although they may influence other uterine leukocytes [25], affect glandular functions [26], modify blood flow directly by acting on spiral arteries [5] or regulate trophoblast invasion [6], their precise functions are unknown.

Mice lacking uNK are fertile but display inadequate uterine vascular remodelling during pregnancy, poor decidualisation [7] and low fetal weight [27,28], highlighting the importance of NK cells in placentation. Despite the differences between mouse and humans in the anatomical details of placentation, length of gestation, time of decidualisation and location of uNK cells, there are also remarkable similarities (Table 1) [1,3,4,7,11,18,29–33]. Notably, NK cells in both species are temporally and spatially associated with trophoblast infiltration into decidua and they are particularly prominent around the spiral arteries [1,34]. In both species, there is a decline in these granulated NK cells from mid-gestation onwards so they are relatively sparse at term [35,36]. Our recent findings also show that like humans, mouse uNK cells express multiple NKR with a repertoire different from peripheral NK cells [32]. Have the NKR on these NK cells become specialised to recognise and respond to ligands on trophoblast cells?

## 3 NK cell receptors

**Fig. 2:**
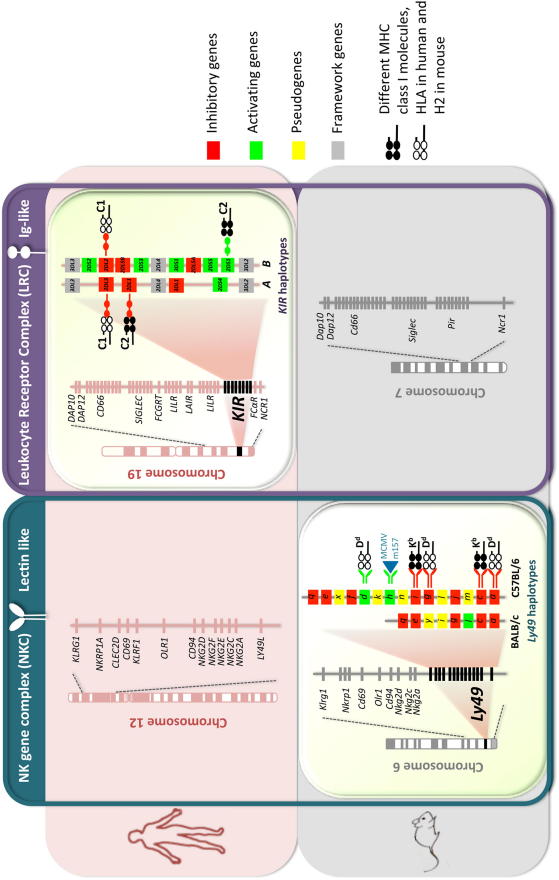
Comparative Genomics of NKC and LRC in human and mouse. A schematic map of the NKC and the LRC encompassing genes encoding for lectin-like and Ig-like NKR and emphasizes the functional homology of human *KIR*[18] and mouse *Ly49*[38] genes that arose by convergent evolution [37]. Two typical human *KIR* haplotypes (*A* and *B*) and mouse *Ly49* haplotypes (BALB/c and C57BL/6) are shown. The MHC class I ligands for the cognate receptors are also depicted for both human and mouse. For clarity, only one MHC class I molecules is indicated for each of the Ly49 receptors, however individual Ly49 receptors can bind multiple MHC class I molecules. For example Ly49C can bind H2-K^b^ and H2-K^d^[39]. The figure is not drawn to scale and not all genes that map to the NKC and the LRC are indicated. Additional information can be found in references [40–43].

NK cell function is controlled by many activating and inhibitory receptors. Structurally, NKR are of two types: Immunoglobulin-like (Ig) receptors (human, killer immunoglobulin-like receptor, KIR) or C-type lectin-like receptors (human and mouse CD94/NKG2 and mouse Ly49). *KIR* genes are clustered in the leukocyte receptor complex (LRC) along with other NKR genes (e.g. *LILR*, *LAIR*, *NCR1*) and genes biologically relevant to pregnancy (e.g. *PSG, CGB, FCRGT*) on human chromosome 19. *CD94/NKG2* genes together with *NKG2D* cluster in the NK gene complex (NKC) on human chromosome 12 and mouse chromosome 6, which also includes Ly49 genes (Fig. 2) [18,37–43].

Contrary to the genes that code for antigen receptors on T and B lymphocytes, the genes coding for NKR do not undergo somatic recombination but are instead germ-line encoded [44]. This means they are subject to natural selection. In the case of human *KIR* genes, balancing selection preserves a high degree of polymorphism in the population, suggesting that no single *KIR* haplotype confers an absolute fitness advantage to an individual [45]. Indeed, recent comparison between human and simian *KIR* genes shows they are rapidly evolving [46]. KIR surface expression is stochastic on individual NK cells [47] and the variegated nature of expression results in many NK cell subsets.

Although the human KIR and mouse Ly49 families are structurally distinct, the intracellular signalling pathways of these receptors are largely conserved [44,48]. Moreover, they represent a remarkable example of convergent evolution as they mediate the same functions in the two species; they bind MHC class I molecules and regulate NK cell function [37]. Both *KIR* and Ly49 gene families are multigenic and polymorphic with different haplotypes having variable gene content including both inhibitory and activating receptors. In addition, both variability in KIR and Ly49 receptors and their MHC ligands determines the outcome of bone marrow transplantation (BMT) further illustrating their homologous functions [49,50]. In mice, the phenomenon of hybrid resistance has been invaluable in illuminating translational research in BMT [51]. Transplantation is, however, an artificial context and the only physiological situation in mammals where NK allo-recognition can occur is during pregnancy. Can murine models therefore unravel the secrets of natural allo-recognition occurring at the maternal-fetal interface in utero?

## 4 NKR on uterine NK cells and trophoblast MHC ligands

Uterine NK cells do express KIR in humans [10,11] and Ly49 in mice [32] as well as a variety of other NKR [4,52,53]. Importantly, the expression of NKR differs in human uNK cells compared to peripheral NK cells: uNK cells are defined as CD56^superbright^ CD16^−^ but unlike their CD56^bright^ peripheral counterparts, they express KIR, NKG2C and NKG2E, as well as CD69, CD117, KLRG1 and CD94 [3,4]. Given the key roles of MHC class I molecules in ontogeny and function of NK cells, MHC expression on trophoblast cells is obviously important to define. In humans, the repertoire of trophoblast HLA class I expression is unique and can be summarised [12]:

i) No expression of HLA-A or HLA-B molecules, which are highly polymorphic and function mainly as T cell ligands.
ii) Expression of oligomorphic non-classical HLA-G and HLA-E molecules, which are ligands for LILR molecules mainly expressed by myeloid cells and the NKR, CD94/NKG2 respectively [54].
iii) Expression of polymorphic classical HLA-C molecules that are the only HLA providing variability and thus may act as a fetal allogeneic ligand depending on the paternal HLA-C.
iv) EVT is the predominant site of HLA-C expression in decidua and paternal HLA-C allotypes are expressed [18].

In collaboration with Myriam Hemberger, we have recently characterised the expression of trophoblast MHC in the C57BL/6 mouse and found [31]:

i) Only one classical MHC class I, H2-K, but not H2-D is expressed.
ii) No non-classical MHC is expressed.
iii) Paternally-inherited H2-K is expressed by trophoblast invading into maternal decidua.

The H2-K molecule was expressed on the surface of trophoblast stem cells (TS) analysed by flow cytometry and by immuno-precipitation of surface proteins. This was confirmed, and intracytoplasmic staining was also seen, when implantation sites at gestation day 8.5 and TS cells were stained for co-localisation of H2-K and cytokeratin or Cdh3 in immunofluorescence microscopy [31].

The parallels, therefore, are that in both humans and mice, MHC expression is selective and differs from other somatic cells. In addition, crucially, we have found paternally inherited MHC antigens are expressed at the maternal-fetal interface in both species so that NK-mediated allo-recognition can potentially occur [18,31].

## 5 HLA-C and KIR

The great diversity of both NKR and MHC genes has been explained by their key roles in the effectiveness of immune responses to pathogens illustrated by a variety of human and mouse studies [55,56]. Our findings now suggest that these immune system genes also affect reproductive success [18,31].

We have a range of evidence to show that there is an important role for NKR and MHC genes in human pregnancy. Because HLA-C is the only trophoblast HLA class I molecule showing any appreciable polymorphism, we investigated expression in uNK cells of those KIR that are devoted to binding HLA-C [11]. We found that:

i) Uterine NK cells express elevated levels of the KIR known to bind HLA-C (KIR2DL1/S1 and KIR2DL2/L3).
ii) HLA-C tetramers bind uNK cells more than peripheral NK cells.
iii) KIR2DL1 and KIR2DS1-Fc fusion proteins bind specifically to surface HLA-C molecules on normal trophoblast [18].
iv) Other groups have found that uNK cell function is altered following ligation of KIR [6].

Given the difficulty in determining exactly how uNK cells might function in different pregnancies where maternal *KIR* and fetal *HLA-C* are both variable, we have used a genetic approach to show how polymorphism of *KIR* and *HLA-C* genes might affect maternal-fetal interactions [15–17]. Genetic epidemiological studies linking certain combinations of human NKR and MHC have driven the biological experiments needed to determine mechanisms of immunity to viruses and we predict a similar development in the field of reproduction. HLA-C ligands for KIR are divided into two groups based on a dimorphism at position 80 of the α1 domain of HLA-C alleles: HLA-C1 (Asn^80^) binds inhibitory KIR2DL2/3 and HLA-C2 (Lys^80^) binds inhibitory KIR2DL1 as well as activating KIR2DS1. The simplest way to analyse *KIR* diversity is to consider all *KIR* haplotypes as either *A* or *B*. *KIR A* haplotypes are simple with 7 genes including only one activating *KIR* (*2DS4*) that is frequently disabled. *KIR B* haplotypes are more variable (<12 genes); these extra genes are mostly activating [45].

We have found that in 3 different pregnancy disorders, all characterised by defective placentation (pre-eclampsia, FGR and RM), the pregnancies most at risk are those where the mother has a *KIR AA* genotype and there is a *HLA-C2* group in the fetus [15–17]. Furthermore, when the frequency of individual *KIR B* haplotypes genes was compared between control and affected women, the genes most reduced in frequency in affected women were at the telomeric end of the *B* haplotype [18]. This is where the activating *KIR* for *HLA-C2* allotypes, *KIR2DS1*, is located. An additional preliminary finding was that fetal *HLA-C2* is a risk factor in *KIR AA* women when it is paternally rather than maternally derived [18]. This risk can be modified by the maternal *C1* or *C2* status in keeping with the ‘rheostat model’, according to which NK cell responsiveness is gradually modulated by inhibitory MHC-NKR interactions during education of the NK receptor repertoire [57]. This suggests that maternal NK cells are educated during their development so that they are calibrated to the levels of maternal *HLA-C*. Thus, uNK responses to the fetus might differ depending on the dose of fetal *HLA-C2* relative to that of the mother’s *HLA-C2* because of the exquisite sensitivity of NK cells to levels of self-MHC. In other words, maternal *C2* genes could modify the risk conferred by fetal *C2* and in keeping with this we find that the *KIR AA* women most at risk are those who are *HLA C1/C1* haplotype carriers confronted by fetal *C2* [18].

## 6 Mouse

The studies in humans reveal the great complexity of NKR-MHC interactions and the need to consider three variables: maternal *HLA-C*, fetal *HLA-C* and maternal *KIR*. Indeed, we have not yet explored the diversity further and considered the contribution of individual alleles of both *HLA-C* and *KIR* (particularly *2DL1*, *2DL2*, *2DL3* and *2DS4* that can all bind subsets of *HLA-C* allotypes [58,59]). Furthermore, the heterogenous nature of these pregnancy disorders and the lack of any easy clear clinical diagnosis of defective placentation makes further progress challenging.

We believe that mouse models can be used to dissect this complexity and determine how individual NKR/MHC combinations influence placentation. The mouse has proved crucial in unravelling how NK cells function in infections [60], BMT [50] and cancer [61]. There are also murine studies (many from Anne Croy’s pioneering work) on the role of uNK cells [62]. Most of the previous work has been conducted in syngeneic mice, however, thus excluding the impact of NKR and MHC variability on placentation.

Recently, we have used both MHC-allogeneic and -congenic mice and shown that paternal MHC affects reproductive outcome [31]. We have also developed robust outcomes of placentation and feto-placental growth, including direct measurements of trophoblast invasion and vascular remodelling. Parameters such as litter size and fetal weight directly determine reproductive success. Evaluation of spiral artery diameter, depth of EVT invasion and placental weight provides insights into indirect effects of individual NKR/MHC combinations. Furthermore, the relatively short life span of mice makes them a suitable model for studies focussing on long-term effects of impaired placentation. For example, insufficient supply of nutrients during fetal development may not have obvious direct effects for the newborn and indeed mice lacking NK cells do produce normal litter sizes. However birth weights are smaller in some NK-deficient strains, such as *Irf1^−/−^* [27] and *Il15^−/−^* mice [28] and thus insufficient nutrient supply during fetal life could prove disadvantageous later in life as it has been shown for the increased risk of hypertension and coronary heart disease in humans [63].

These findings and experimental protocols will allow future experiments where genetically modified mice, for example congenic for NKR, lacking H2-K or expressing known NK ligands as transgenes can be used. Additional questions relating to uNK cell memory and uNK cell education by maternal and fetal MHC can also be addressed in a systematic way. The phenomenon of NK cell memory [64], where NK cells respond more readily to the same insult on secondary encounter has obvious relevance to human pregnancy - the risk of pre-eclampsia is reduced after a successful first pregnancy but this effect is lost after long interbirth intervals [65].

## 7 Future directions

Although our initial genetic studies have revealed that particular combinations of NKR *KIR* and MHC *HLA-C* genotypes are associated with an increased risk of pregnancy disorders, much further work at both genetic and functional levels is required before these findings can be translated to the clinic. Firstly, because we have only genotyped British people of European descent to date, similar genetic studies must be performed in other populations. Because of the high incidence of pre-eclampsia in Africans, this will be a particularly informative population to study.

Furthermore, we have only performed basic *KIR* and *HLA* genotyping and not considered allelic variation. In the context of hepatitis C virus infections, where *HLA-C1/C1* haplotype carriers homozygous for *KIR2DL3* have a higher probability of resolution [66], certain *HLA-C* alleles are now emerging as particularly beneficial [67]. Those KIR that can potentially bind HLA-C2 allotypes (2DL1, 2DL2, 2DS4 and 2DS1) also show some allelic variation and this is likely to be functionally important. Future prospective studies with large numbers of well-characterised pregnancies and information on uterine artery blood flow are needed before any definitive conclusions can be reached about ‘good’ or ‘bad’ *KIR/HLA-C* combinations in humans. These will need to be supported by functional assays using uNK cells isolated from first trimester decidua and experiments using mouse models with defined NKR/MHC combinations. Nevertheless, the findings to date do indicate that immune system genes are likely to be important in reproductive success. Interestingly, a powerful and diverse immune system comes with a price for the individual [68] and a trade-off between its function and reproductive success has been suggested in humans [69] and described in other species such as Soay sheep [70].

## Figures and Tables

**Table 1:** Comparison of the maternal-fetal interface in humans and mice.

Feature	Human	Mouse
Similarities in placenta type	Discoidal, chorio-allantoic [29]	Discoidal, chorio-allantoic [29]

Haemochorial placentation	Yes	Yes

Decidualised endometrium	Yes, during each menstrual cycle [29]	Yes, triggered by implantation [29]

Disproportionate feto-placental growth	Fetal growth disproportionally higher compared with placental growth in late gestation [29]	Fetal growth disproportionally higher compared with placental growth in late gestation [29]

Syncytial transport and barrier trophoblast	Chorionic villi with syncytiotrophoblast barrier formed by cell fusion. Diploid nuclear DNA content [30].	Labyrinth with syncytiotrophoblast barrier formed by cell fusion. Diploid nuclear DNA content [30]


Invasive trophoblast	Non-proliferative, mononuclear polyploid cytotrophoblast [30]. Invasion of decidua basalis and inner third of the myometrium and differentiation into trophoblast giant cells. EVT face maternal immune system [29].	Non-proliferative, mononuclear polyploid trophoblast with giant cells [30]. Invasion of decidua basalis. Trophoblast giant cells face maternal immune system [29]

Selective MHC class I expression by trophoblast	HLA-C, HLA-G and HLA-E; no HLA-A or HLA-B [12]	H2-K; no H2-D or Qa1 (in C57BL/6 mice [31])

Unique uNK phenotype	CD69^+^ CD117^+^/-KLRG1^high^ CD56^superbright^CD9^+^CD94^bright^[3, 4]	CD69^+^ CD117^+^ KLRG1^high^ NK1.1-DX5- [32]^a^


Activating NKR on uNK cells	NKG2D, NKp46, KIR2DS1 [6, 18]	NKG2D, NKp46, Ly49H, CD16 [32]


Inhibitory NKR on uNK cells	KLRG1, KIR2DL1/2/3, NKG2A [3, 6]	KLRG1, Ly49A, Ly49C, Ly49G2 [31, 32]

Vascular changes concomitant with uNK infiltration of uterus	uNK cells accumulate at site of placentation during the first trimester concentrating around spiral arteries [1]	High degree of uNK cell infiltration at gestational day 9.5 [30] around the spiral artery [7]

Highly polymorphic receptor/ligand systems	KIR on uNK/HLA-C on EVT [18]	Ly49s on uNK/H2-K on trophoblast giant cells [31, 32]

Expression of paternal MHC	Yes [18]	Yes [31]

Impact of paternal MHC on reproductive success	Combination of maternal *KIR-AA* and fetal *HLA-C2* genotypes increases the risk for pre-eclampsia, FGR and RM [15,17,18]	Antigenic disparity between parental *H2-*linked genes affects transformation of the uterine vasculature, as well as fetal growth and placental efficiency [31]

a A different immunophenotype of decidual NK cells in C57BL/6 mice was reported by Mallidi et al., although the cells analysed in this study were mostly DBA- NK1.1^+^[31].
